# BAM-STR: A Bio-Inspired Soft Tensegrity Robot Driven by McKibben Pneumatic Artificial Muscles

**DOI:** 10.3390/mi17070857

**Published:** 2026-07-17

**Authors:** Yang Jiang, Xinyuan Yang, Zihao Zuo, Yunkai Chen, Shizhuo Zhang, Hong Jiang, Shaojie Gu, Yanhong Peng

**Affiliations:** 1 School of Mechanical Engineering, Chongqing University of Technology, Banan, Chongqing 400054, China; 2 Magnesium Research Center, Kumamoto University, Kumamoto 860-8555, Japan; 3 Faculty of Advanced Science and Technology, Kumamoto University, Kumamoto 860-8555, Japan

**Keywords:** soft tensegrity robot, McKibben pneumatic artificial muscle, peristaltic crawling, bio-inspired locomotion

## Abstract

Tensegrity structures have lightweight, compliant, impact-resistant, and large-deformation characteristics, providing a deformable structural solution for mobile robots in complex environments. Inspired by earthworm peristaltic locomotion, this study proposes BAM-STR, a soft tensegrity robot driven by McKibben pneumatic artificial muscles. The robot adopts a three-layer, three-strut tensegrity structure, and the McKibben pneumatic artificial muscles are arranged at the diagonal and additional tendon positions to generate axial–radial coupled deformation under low-pressure actuation. A bio-inspired segmented peristaltic waveform control strategy is further designed. By sequentially activating and releasing the artificial muscles in the three tensegrity units, the robot generates an axially propagating deformation wave and achieves continuous forward crawling. Experimental results show that BAM-STR can achieve approximately 31% axial contraction and 21% radial expansion at an input pressure of 100kPa. When the control time interval is ΔT=1.0–1.25s, the robot reaches its maximum average crawling speed of approximately 6.5mm/s. Multi-scenario experiments further show that BAM-STR can adapt to channel widths ranging from 190 to 235mm, complete continuous crawling while carrying an additional payload of 200g, and maintain forward locomotion on a rough artificial grass surface. These results indicate that BAM-STR has path-width adaptability, load-carrying crawling capability, and rough-ground adaptability.

## 1. Introduction

Robots have important application value in unknown-space exploration, disaster rescue, pipeline inspection, and locomotion across complex terrains [1,2,3,4,5,6]. In these scenarios, robots usually need to operate in narrow passages, irregular obstacle distributions, and complex contact conditions [7]. Conventional rigid robots may suffer from collision, jamming, and posture instability when moving through narrow, rugged, or unpredictable spaces [8,9,10,11]. Therefore, lightweight, compliant, and environmentally adaptive mobile robots have become an important research direction [12,13,14,15,16].

Tensegrity structures provide a promising structural solution for compliant robot design. A tensegrity structure is generally composed of discrete compressive members and continuous tensile members, and its stable configuration is maintained by internal prestress [17,18,19,20,21,22]. Since the compressive members are usually not in direct contact, external loads can be redistributed through the cable–strut network, giving the structure lightweight, compliant, impact-resistant, and large-deformation characteristics [23,24,25]. These properties make tensegrity structures attractive for mobile robots working in complex environments.

Several single-unit tensegrity robots have been developed based on six-strut or similar tensegrity configurations. Kim et al. [26], Hao et al. [27], Vespignani et al. [28], and Doney et al. [29] realized different rolling or translational behaviors by using linear actuators, electric push rods, motor-driven cable spooling, or vibration motors. These studies demonstrate the feasibility of tensegrity structures for impact-resistant and deformable mobile robots. However, such robots usually rely on local structural deformation to shift the center of gravity and then use self-weight to complete rolling motion. This locomotion mechanism requires sufficient flipping space and is easily constrained in narrow passages, pipelines, caves, and cluttered environments. In addition, rigid actuators and cable-spooling mechanisms increase system mass and structural complexity, which may affect locomotion stability and control accuracy.

To overcome the limitations of single-unit rolling robots, researchers have developed crawling robots composed of multiple tensegrity units. Liu et al. [30] proposed a tensegrity-based inchworm-like robot for crawling in pipes with varying diameters. Böhm et al. [31] designed a worm-like mobile robot based on tensegrity structures, enabling local extension, gripping, and bidirectional peristaltic crawling. Zappetti et al. [32] proposed a bio-inspired modular tensegrity robot that generates peristaltic locomotion through sequential tendon contraction. Compared with single-unit rolling robots, these multi-unit crawling robots do not rely on whole-body tumbling and are more suitable for confined spaces. Although multi-unit tensegrity crawling robots show promising locomotion potential, their rigid actuation systems still limit structural deformation. Motor-driven actuators, pulleys, and cable-winding mechanisms often occupy the internal space of each module, restricting contraction and folding. Their added volume and mass can also reduce locomotion efficiency and environmental adaptability.

To address this issue, soft actuators have been introduced into tensegrity robot design. Common soft actuation methods include shape memory alloy (SMA) and pneumatic artificial muscle (PAM). Chung et al. [33] developed a tensegrity robot based on torsionally prestrained SMA springs and realized rolling and jumping motions. Kazoleas et al. [34] proposed tensiworm, an earthworm-inspired tensegrity robot composed of three serially connected icosahedral tensegrity units, in which SMA active cables are used to generate segmented peristaltic locomotion. However, SMA actuation depends on electrothermal conversion and material phase transformation, so its response speed and actuation frequency are easily limited by heating and cooling processes.

In addition to SMA, PAM have been widely used in soft robotic and haptic assistance systems due to their compliance, lightweight structure, and muscle-like contraction characteristics [35,36]. They have also been applied in tensegrity robot research to drive active structural deformation and bio-inspired locomotion. Li et al. [37] combined thin PAM, a three-strut tensegrity structure, and soft sensor threads to construct a deformable tensegrity unit for shape recognition. Kobayashi et al. [38] further developed a soft tensegrity robot driven by thin artificial muscles for exploring unknown spatial configurations. By connecting five tensegrity units in series, the robot achieved inchworm-like locomotion between walls, through curved paths, and in vertical pipes. However, thin PAM usually require relatively high input pressure to approach their maximum contraction performance, and their fabrication and assembly are relatively delicate, leading to higher manufacturing cost.

**Figure 1:**
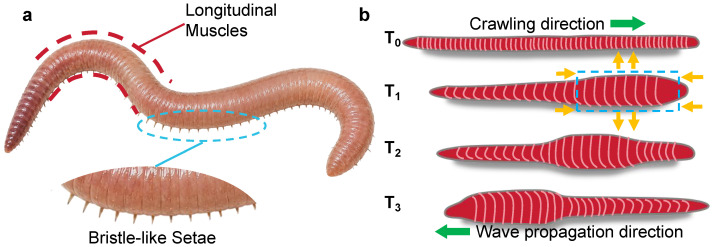
Bio-inspired design inspiration of BAM-STR based on earthworm locomotion. (**a**) Key locomotion-related structures of an earthworm, including longitudinal muscles and bristle-like setae. The longitudinal muscles contribute to body deformation, while the setae provide anchoring and traction during ground contact. (**b**) Schematic illustration of earthworm-like peristaltic crawling. Local axial contraction and radial expansion generate a propagating peristaltic wave, which enables forward crawling through frictional interaction with the ground.

To address the above challenges, this study proposes BAM-STR, a soft tensegrity robot driven by PAM. The structural design and motion control strategy of BAM-STR are inspired by the locomotion mechanism of earthworms, as shown in Figure 1. Earthworms achieve local body extension and contraction through coordinated muscle contraction, while their bristle-like setae provide anchoring and frictional interaction with the ground. Inspired by this mechanism, the robot adopts a three-layer, three-strut tensegrity structure as the main framework, and the fabricated PAM are arranged at the diagonal and additional tendon positions. Experimental results show that BAM-STR can achieve approximately 31% axial contraction and 21% radial expansion at an input pressure of 100kPa. A bio-inspired segmented peristaltic waveform control strategy is also proposed. This strategy is inspired by the peristaltic locomotion mechanism of earthworms, as shown in Figure 1b. By sequentially activating and releasing the artificial muscles in the three tensegrity units, the robot can generate an axially propagating deformation wave and achieve continuous forward crawling. Experimental results show that the control time interval ΔT has a clear influence on the crawling speed. When ΔT=1.0–1.25s, the robot reaches its maximum average crawling speed of approximately 6.5mm/s. Finally, multi-scenario experiments were conducted to evaluate the crawling adaptability and motion stability of BAM-STR. The results show that the robot can adapt to channel widths ranging from 190 to 235mm. It can also complete a continuous crawling distance of 600mm while carrying an additional payload of 200g, with an average speed of 2.59mm/s. Moreover, the robot can maintain continuous crawling on a rough artificial grass surface, with an average speed of 1.75mm/s. These results indicate that BAM-STR has a certain degree of path-width adaptability, load-carrying crawling capability, and rough-ground adaptability, providing an experimental basis for the application of soft tensegrity robots in confined spaces and complex ground environments.

## 2. Structural Design and Fabrication of BAM-STR

### 2.1. Parametric Design of the Three-Layer Three-Strut Tensegrity Structure

The proposed robot adopts a three-layer, three-strut tensegrity structure, which is assembled by connecting three basic three-strut units along the axial direction. The structure consists of struts and strings. The struts serve as compressive members, while the strings serve as tensile members. According to their functions, the strings are further divided into horizontal strings, saddle strings, additional strings, and diagonal strings. The horizontal strings are used to maintain the triangular profile of each layer. The saddle strings connect adjacent unit interfaces. The diagonal strings are arranged inside each basic unit to maintain the internal torsional stability. The additional strings connect adjacent units and provide axial connection between the units. In total, the structure contains 18 nodes and 48 members, including 9 struts and 39 strings.

Each member is defined by its start and end nodes, and all connection relationships are assembled into a member connectivity matrix for the subsequent equilibrium matrix construction. Based on the SVD-based form-finding method reported by Luo et al. [39], the self-equilibrated configuration of the proposed structure is determined by introducing force density, constructing the equilibrium matrix, and using the minimum singular value to identify feasible self-stress states.

A self-equilibrated tensegrity configuration needs to satisfy the force balance condition. Following the force-density formulation, the force density of an arbitrary member *e* is defined as

(1) $$q_{e}=\frac{F_{e}}{l_{e}},$$

where $F_{e}$ is the axial force of the member and $l_{e}$ is the member length. For an ideal tensegrity structure, the struts should be in compression, while the strings or pneumatic artificial muscles should be in tension. Therefore, the force density signs should satisfy

(2) $$q_{\mathrm{bar}}<0,\;q_{\mathrm{string}}>0.$$

When an arbitrary node in the structure is in static equilibrium, the vector sum of the axial forces of all members connected to this node should be zero, namely

(3) $$\sum q_{ij}\left(n_{j}-n_{i}\right)=0,$$

where $n_{i}$ and $n_{j}$ are the position vectors of the two connected nodes, and $q_{ij}$ is the force density of the member connecting nodes *i* and *j*. By assembling the equilibrium equations of all nodes, the global equilibrium equation can be expressed as

(4) Eq=0,

where *E* is the equilibrium matrix and q is the vector composed of the force densities of all members. If this equation has a non-trivial solution, the structure has a self-stress state.

To obtain the self-stress distribution of the structure, singular value decomposition is performed on the equilibrium matrix *E*. When the minimum singular value is sufficiently close to zero, the equilibrium matrix possesses an approximate null space. The right singular vector corresponding to the minimum singular value is then taken as the force-density solution of the structure. The resulting solution is further examined to verify that the struts and strings satisfy the required sign conditions.

**Figure 2:**
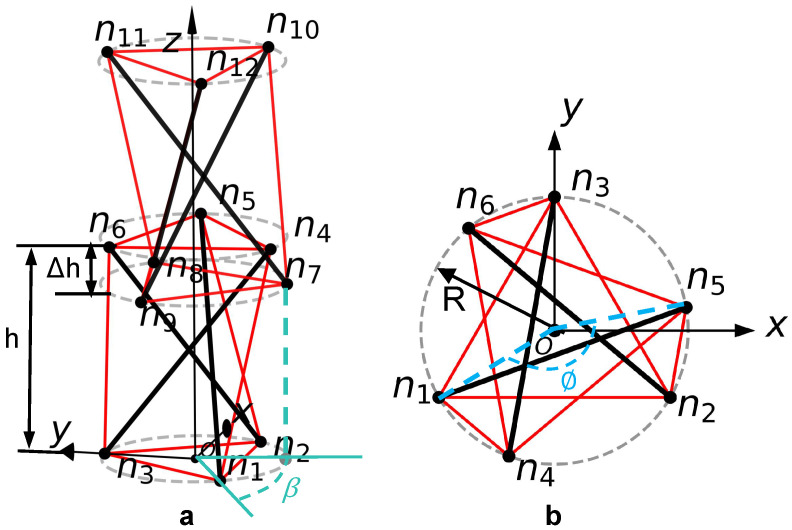
Geometric parameters of the tensegrity structure. (**a**) Unit assembly and the parameters *h*, Δh, and β, where k=Δh/h. (**b**) Top view showing *R* and ϕ.

To describe the spatial configuration of the three-layer, three-strut tensegrity structure, the structural radius *R*, unit length *h*, unit twist angle ϕ, inter-unit rotation angle β, and overlap ratio *k* are introduced, as shown in Figure 2. Figure 2a illustrates the assembled configuration of adjacent units, where Δh denotes the axial overlap length between adjacent units, the overlap ratio is defined as k=Δh/h, and β denotes the angle between the lines connecting the origin to the initial nodes on the lower faces of the upper and lower units. Figure 2b shows the top view of a unit, where *R* denotes the radius of the circumscribed circle of the end face, and ϕ denotes the unit twist angle. By adjusting ϕ, β, and *k*, the overall spatial configuration of the structure can be varied.

**Figure 3:**
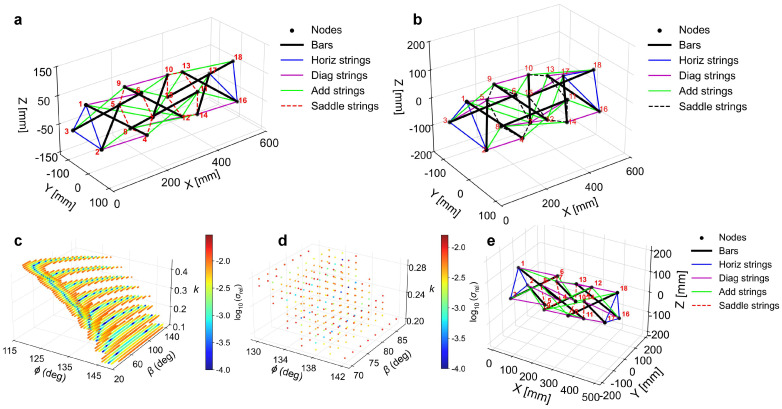
Parametric design and self-equilibrated configuration screening of the three-layer, three-strut tensegrity structure. (**a**) Boundary configuration at k=0. (**b**) Boundary configuration at k=0.5. (**c**) Coarse scanning results in the global parameter space ϕ∈[0°,180°], β∈[0°,180°], and k∈(0,0.5). (**d**) Fine scanning results in the stable region ϕ∈[130°,150°], β∈[70°,90°], and k∈[0.2,0.3]. (**e**) Structural topology model with R=80mm, h=180mm, ϕ=140°, β=80°, and k=0.2.

To avoid structural degeneration and ensure a reasonable axial overlap between adjacent units, the overlap ratio *k* is set within the range of 0<k<0.5. When k=0, adjacent units are directly stacked without any axial overlap, as shown in Figure 3a. When k=0.5, the upper nodes of the first layer overlap with the lower nodes of the third layer, leading to geometric degeneration, as shown in Figure 3b.

A parameter scan was then performed using Python over the ranges of ϕ∈[0°,180°], β∈[0°,180°], and k∈(0,0.5). According to the coarse scanning results shown in Figure 3c, the stable solutions of the three-layer, three-strut tensegrity structure are not uniformly distributed over the entire parameter space, but are concentrated in a band-shaped region. In this region, the point density is relatively high within ϕ∈[130°,150°], and the corresponding values of $\sigma_{\mathrm{rel}}$ are relatively small. This indicates that the structure is closer to an equilibrium state in this interval and exhibits better stability. Therefore, ϕ∈[130°,150°], β∈[70°,90°], and k∈[0.2,0.3] were selected as the fine scanning ranges for further analysis. The fine scanning results are shown in Figure 3d. In this parameter space, the stable solutions satisfying the self-equilibrium condition are continuously distributed, indicating that this region can be regarded as an effective parameter space for stable configurations. Further analysis shows that parameter combinations with better stability are mainly concentrated in the middle of this region. When k=0.2, the corresponding relative singular value $\sigma_{\mathrm{rel}}$ is relatively small, indicating that the configuration is close to an equilibrium state. Based on the above results, ϕ=140°, β=80°, and k=0.2 are selected as the representative parameter combination. Further analysis shows that parameter combinations with better stability are mainly concentrated in the middle of this region. When k=0.2, the corresponding relative singular value $\sigma_{\mathrm{rel}}$ is relatively small, indicating that the configuration is close to an equilibrium state. Based on the above results, ϕ=140°, β=80°, and k=0.2 are selected as the representative parameter combination. This combination is located inside the stable configuration region rather than near its boundary, suggesting that the SVD-based form-finding result has a certain tolerance to small deviations in the initial geometric parameters.

Meanwhile, the node distribution radius and the unit height are set to R=80mm and h=180mm, respectively. Here, *R* determines the radial size of the structure, while *h* determines the axial height of a basic unit. These scale parameters are determined according to the installation space of the artificial muscles, the strut-end connection method, and the overall size of the experimental prototype. They are used as fixed dimensional conditions for the subsequent configuration design and parameter scanning.

In summary, the final parameters of the three-layer, three-strut tensegrity structure are determined as R=80mm, h=180mm, ϕ=140°, β=80°, and k=0.2, and configuration verification is then performed based on this parameter set. As shown in Figure 3e, the three-layer, three-strut units form a stable twisted and overlapped spatial configuration. The saddle strings, additional strings, and diagonal strings establish continuous force transmission paths between adjacent units, giving the overall structure good spatial integrity and self-equilibrium characteristics. Based on the selected parameter combination, the lengths of different structural members are further calculated, as summarized in Table 1. These results provide dimensional references for the subsequent prototype fabrication and artificial muscle arrangement.

### 2.2. Prototype Fabrication and Component Integration

**Figure 4:**
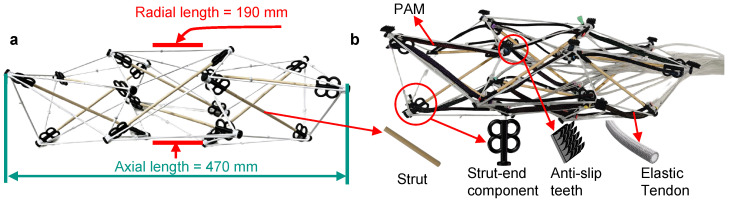
BAM-STR prototype design and component layout. (**a**) Three-layer, three-strut tensegrity rod–tendon prototype without artificial muscles. (**b**) Actuated prototype after installing PAMs and key components, including struts, strut-end components, anti-slip teeth, elastic tendons, and PAMs. The prototype has an axial length of approximately 470mm and a radial length of approximately 190mm.

Based on the selected configuration parameters, the prototype fabrication and component integration of the three-layer, three-strut tensegrity robot were completed. The prototype consists of lightweight wooden struts, elastic tendons, strut-end components, and PAMs. The wooden struts serve as compressive members to support the overall spatial shape, while the elastic tendons serve as tensile members to establish the prestress network and maintain structural stability. According to the design parameters, the prototype has an axial length of approximately 470mm and a radial length of approximately 190mm, as shown in Figure 4a.

To satisfy the requirements of structural connection and ground support, a strut-end component was installed at each end of the struts. This component connects the struts, elastic tendons, and PAMs, and also serves as the main supporting part during the ground contact phase. To enhance the frictional constraint during crawling, anti-slip teeth were designed at the bottom of the strut-end component. The anti-slip structure consists of multiple inclined tooth-like elements, which can generate different frictional resistance in the forward and backward directions. This anisotropic friction helps reduce backward slipping and improve the propulsion efficiency of the robot. To achieve active structural deformation, PAMs were arranged at selected diagonal and additional tendon positions. The complete robot prototype is shown in Figure 4b.

The PAMs were fabricated based on our previous work [40]. A PET flame-retardant nylon braided sleeve was used as the constraint layer. The sleeve had a flat geometry with widths of 6mm and 10mm, a braiding angle of less than 25°, and a diameter expansion ratio of approximately 1.5. A thickened latex balloon was used as the inflatable chamber, and a polyethylene (PE) tube with an outer diameter of 4mm and an inner diameter of 2mm was used as the air inlet tube. During fabrication, the distal end of the artificial muscle was sealed using cable ties and adhesive, while the inlet tube was inserted into the latex balloon and fixed with cable ties to ensure airtightness and connection reliability.

**Figure 5:**
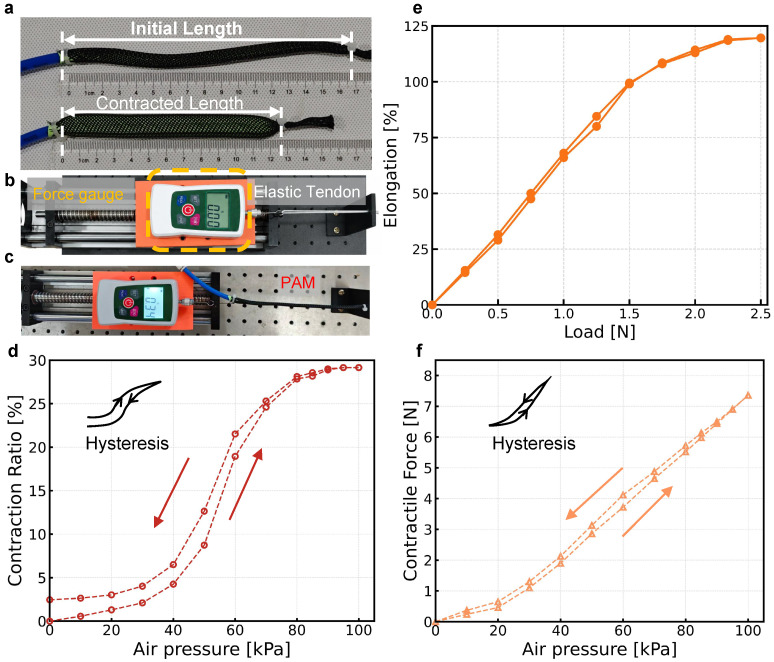
Experimental characterization of the elastic tendon and PAM. (**a**) Comparison between the initial length and contracted length of the artificial muscle. (**b**) Tensile testing platform for the elastic tendon. (**c**) Output force testing platform for the artificial muscle. (**d**) Pressure–contraction ratio curve of the artificial muscle. (**e**) Load–elongation ratio curve of the elastic tendon. (**f**) Pressure–contraction force curve of the artificial muscle.

Subsequently, the mechanical characteristics of the PAMs and elastic tendons used in the structure were experimentally evaluated, and the corresponding experimental setups are shown in Figure 5a–c. The experimental results show that the fabricated PAMs produced obvious axial contraction as the input pressure increased. At an input pressure of 100kPa, the contraction ratio reached approximately 29%, as shown in Figure 5d. Meanwhile, obvious hysteresis was observed during cyclic pressurization and depressurization. This behavior is mainly related to the nonlinear deformation and friction among the latex balloon, braided constraint layer, and end-fixing structure.

The elastic tendons act as tensile members in the tensegrity structure, and their tensile response directly affects the prestress distribution and self-equilibrium stability of the robot. Therefore, the load–elongation ratio relationship of the elastic tendons was measured, as shown in Figure 5e. The results show that the selected elastic tendons exhibit high extensibility, reaching an elongation ratio of approximately 120% under a tensile load of about 2.5N. This large elongation under a relatively low tensile load allows the tendons to maintain the initial tension state while providing sufficient extension for PAM-driven structural deformation, thereby balancing the requirements of structural pretension and overall compliance.

In addition, the fabricated PAMs generated a relatively large contraction force. At an input pressure of 100kPa, the measured contraction force was approximately 7.4N, as shown in Figure 5f. The contraction-force response also exhibited obvious hysteresis during cyclic pressurization and depressurization.

**Figure 6:**
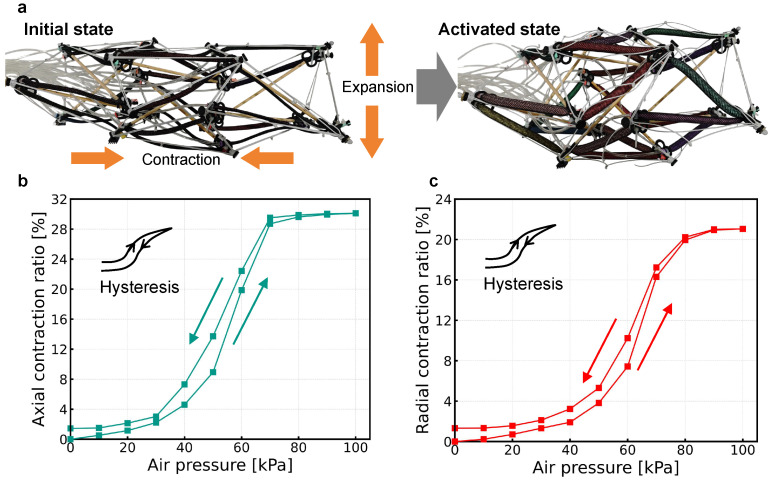
Axial–radial coupled deformation of BAM-STR under synchronous actuation. (**a**) Morphological change of the robot before and after synchronous pressurization of all artificial muscles. (**b**) Axial contraction ratio under different input pressures. (**c**) Radial expansion ratio under different input pressures.

To evaluate the overall deformation capability of BAM-STR under standard actuation conditions, all artificial muscles were synchronously pressurized in this experiment. As a result, the robot exhibited obvious three-dimensional deformation, mainly characterized by axial contraction and radial expansion, as shown in Figure 6a. As the input pressure increased, the axial contraction ratio of the robot gradually increased and reached approximately 31% at 100kPa, as shown in Figure 6b. Meanwhile, the radial expansion ratio also increased with the input pressure and reached approximately 21% at 100kPa, as shown in Figure 6c. During depressurization, both the axial contraction ratio and radial expansion ratio did not fully follow the pressurization paths, indicating a certain hysteresis behavior. This experiment demonstrates that the proposed three-layer, three-strut structure can stably generate axial–radial coupled deformation under the standard actuation pressure, which provides the deformation basis for subsequent peristaltic crawling experiments.

## 3. Control System

**Figure 7:**
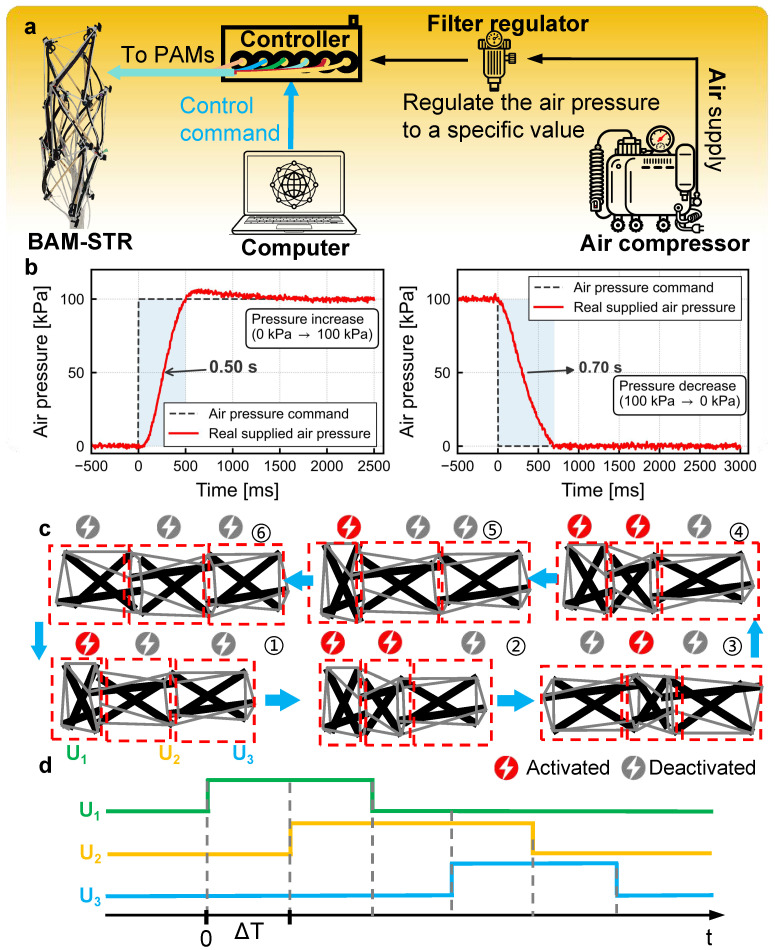
Pneumatic control system and timing control strategy of BAM-STR. (**a**) Pneumatic control system, including an air compressor, a filter regulator, a multi-channel pressure controller, and a host computer. (**b**) Pressure response curve of the pneumatic system. (**c**) Bio-inspired peristaltic segmented waveform control logic in which the three units $U_{1}$, $U_{2}$, and $U_{3}$ are sequentially activated and released to form an axially propagating deformation wave. (**d**) Corresponding timing control signals of the three units, where ΔT denotes the time interval between two adjacent control actions.

The pneumatic control system of BAM-STR is composed of an air compressor, a filter regulator, a customized multi-channel pressure controller (S-HCDW-R1), and a host computer, as shown in Figure 7a. The air compressor provides the pneumatic source, while the filter regulator stabilizes the input pressure. The controller regulates the output pressure of each channel according to the commands sent from the host computer. The control program was developed using Python 3.10 and executed on the control computer.

The proposed robot consists of three three-strut tensegrity units. In each unit, the PAMs arranged at the diagonal tendon positions and those arranged at the additional tendon positions are controlled by two independent pneumatic channels. Therefore, the entire robot uses six independent pneumatic channels. Considering the contraction efficiency of the PAMs and the structural stability of the robot, the maximum operating pressure was set to 100kPa. To evaluate the response performance of the pneumatic system, the pressure response was tested between 0kPa and 100kPa, as shown in Figure 7b. The results indicate that the system can rapidly establish and release the target pressure. Specifically, the pressure increased from the baseline to 100kPa within approximately 0.5s, and decreased back to the baseline within approximately 0.7s.

To generate peristaltic crawling, the three tensegrity units are sequentially defined as $U_{1}$, $U_{2}$, and $U_{3}$ along the axial direction, as illustrated in Figure 7c. In this study, a bio-inspired peristaltic waveform control strategy is adopted, which is inspired by the periodic peristaltic locomotion of soft-bodied animals such as earthworms. In the standard peristaltic control mode, each unit is regarded as an independent actuation segment. When a unit is activated, the PAMs arranged at both the diagonal and additional tendon positions inside this unit are simultaneously pressurized and contracted, resulting in local contraction and posture variation of the unit. By sequentially activating and releasing $U_{1}$, $U_{2}$, and $U_{3}$, a deformation wave propagates along the axial direction of the robot. Together with the directional friction provided by the anti-slip teeth at the strut ends, this propagating deformation wave enables forward peristaltic crawling.

Although the experimental platform provides two independent pneumatic channels for each unit, which separately control the PAMs at the diagonal and additional tendon positions, the two types of PAMs in the same unit are synchronously activated in the standard peristaltic gait. Therefore, the basic peristaltic motion can be equivalently regarded as a three-segment sequential actuation process. The six-channel design retains the capability of independently controlling the diagonal and additional PAMs, which provides an experimental basis for further investigating the influence of different muscle combinations on locomotion performance.

The corresponding timing control signals of the three units are shown in Figure 7d. The time interval between two adjacent control actions is defined as ΔT. By adjusting ΔT, the propagation speed of the deformation wave among the three units can be changed, thereby regulating the overall crawling speed of the robot.

## 4. Experimental Evaluation

### 4.1. Basic Peristaltic Crawling and Timing-Interval Optimization

**Figure 8:**
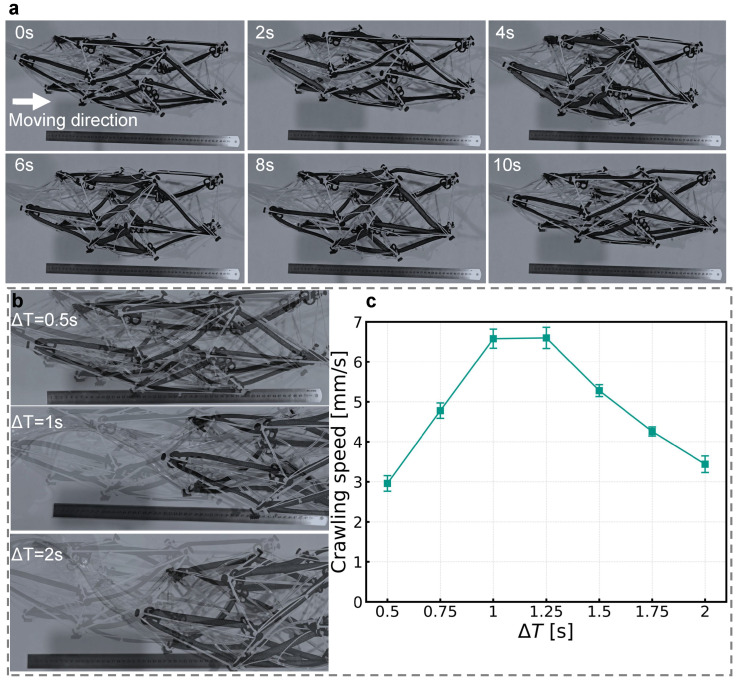
Basic peristaltic crawling experiment of BAM-STR and timing-interval optimization. (**a**) Crawling process of the robot within a full gait cycle when ΔT=2s. (**b**) Comparison of the robot’s motion under different ΔT conditions. (**c**) Effect of different ΔT values on the average crawling speed. The results show that the crawling speed initially increases and then decreases as ΔT increases, reaching an optimal range at ΔT=1.0–1.25s.

To verify the effectiveness of the proposed bio-inspired peristaltic waveform control strategy, a basic crawling experiment was first conducted on BAM-STR. The time interval between consecutive control actions ΔT was set to 2s to ensure that each unit could complete sufficient deformation. The six sequential control stages under this timing condition are illustrated in Figure 8a. Within a complete gait cycle, BAM-STR advanced approximately 35mm along the forward direction, corresponding to an average crawling speed of about 3.5mm/s. These results demonstrate that the designed timing control strategy effectively coordinates the contraction–release cycles of each unit, enabling continuous and controllable peristaltic locomotion.

To investigate the influence of timing intervals on crawling performance, the robot’s average speed was measured under various ΔT values, as shown in Figure 8b,c). The results indicate that ΔT has a significant effect on crawling speed. When ΔT is short, the driving frequency is high, but the muscles in each unit have insufficient time to fully contract, resulting in smaller per-cycle displacements and, consequently, lower average speeds. As ΔT increases, the units can undergo more complete contraction and release, and the crawling speed gradually rises. The highest speeds, around 6.5 mm/s, are achieved at ΔT=1.0–1.25s. Further increasing ΔT allows for more complete unit deformation per cycle but reduces the number of cycles completed per unit time, thereby lowering the average speed. These results indicate that an intermediate timing interval balances unit deformation amplitude and gait cycle duration, optimizing the peristaltic crawling efficiency of BAM-STR.

### 4.2. Effect of Active Tendon Actuation Modes on Crawling Performance

**Figure 9:**
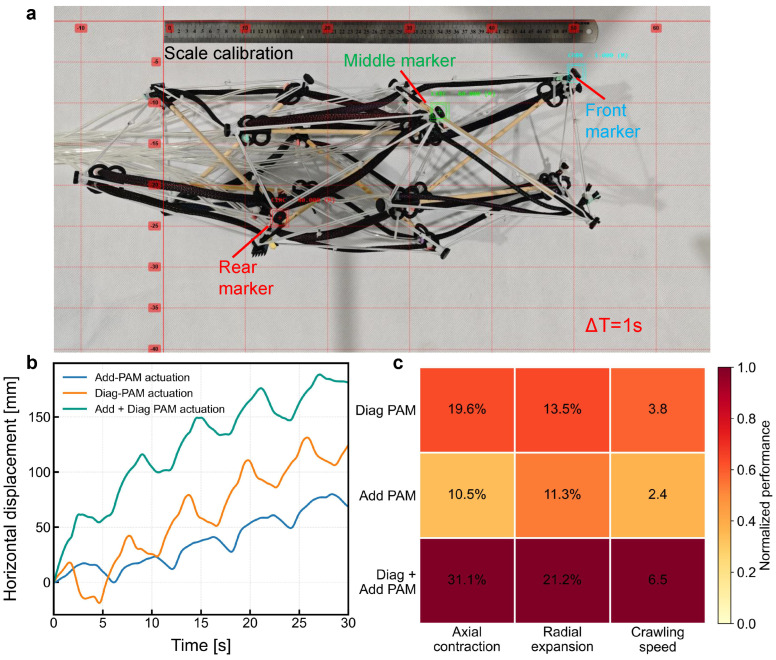
Peristaltic performance evaluation of BAM-STR under different active tendon actuation modes. (**a**) Experimental setup and marker arrangement for tracking the robot motion. (**b**) Average horizontal displacement of the robot over time under different actuation modes, with ΔT=1s. (**c**) Normalized contribution heatmap of different active tendon actuation modes to the axial contraction ratio, radial expansion ratio, and crawling speed, used to compare their effects on structural deformation and locomotion performance.

To investigate the contribution of different active tendon groups to the peristaltic crawling performance of BAM-STR, three actuation modes were compared: actuation of only the additional tendon PAMs, actuation of only the diagonal tendon PAMs, and synchronous actuation of both additional and diagonal tendon PAMs. In all experiments, the control interval was fixed at ΔT=1s, and each actuation mode was continuously executed for 30s. The robot’s motion was recorded using a fixed camera and analyzed with Kinovea software (version 2025.2). Prior to the experiments, the scale was calibrated using a ruler visible in the images, and one prominent marker was selected on each of the three units as a tracking point, as shown in Figure 9a. By recording the horizontal displacement of the three markers and calculating their mean value, the overall horizontal displacement of the robot over time was obtained, as shown in Figure 9b.

The results indicate that the active tendon actuation mode has a significant effect on the crawling performance of BAM-STR. When the additional and diagonal tendon PAMs were synchronously actuated, the robot achieved a considerably larger horizontal displacement within 30s than when either tendon group was actuated individually. This result suggests a strong cooperative effect between the two tendon groups. Such synergy enhances the axial–radial coupled deformation of the structure, increases the effective displacement per gait cycle, and thereby improves the crawling speed.

Furthermore, when only the additional tendon PAMs or only the diagonal tendon PAMs were actuated, BAM-STR was still able to achieve continuous forward displacement, indicating that a single tendon system also provides independent propulsion capability. These findings demonstrate that BAM-STR’s peristaltic motion does not entirely depend on a specific tendon type, and the structure retains a degree of redundancy and fault tolerance in the event of partial actuator failure. Figure 9c further presents a normalized contribution heatmap of the axial contraction ratio, radial expansion ratio, and crawling speed under different actuation modes, providing a visual comparison of the effects of various active tendon combinations on structural deformation and locomotion performance.

### 4.3. Multi-Scenario Crawling Adaptability Tests

To further evaluate the environmental adaptability of BAM-STR, three crawling experiments were conducted under different operating conditions, including confined channels with different path widths, flat-surface crawling with additional payload, and crawling on an artificial grass surface.

#### 4.3.1. Path-Width Adaptability in Confined Channels

**Figure 10:**
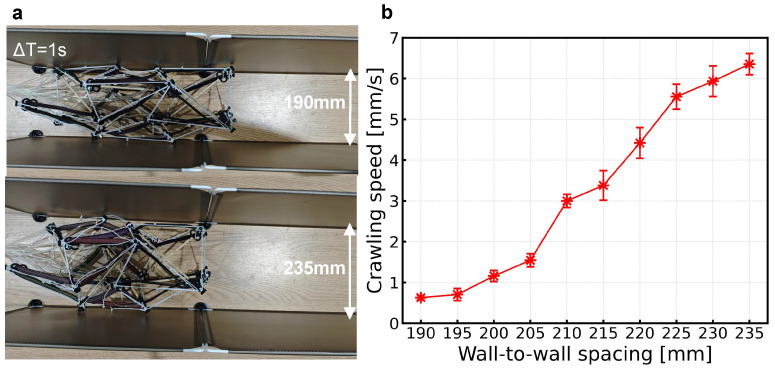
Crawling adaptability test of BAM-STR under different path widths, with the control interval fixed at ΔT=1s. (**a**) Representative experimental scenes at path widths of 190mm and 235mm. (**b**) Variation in average crawling speed as the path width increases from 190mm to 235mm with a step size of 5mm.

For robots operating in confined environments, adaptability to different path widths is an important factor affecting locomotion performance. Therefore, the path-width adaptability of BAM-STR was evaluated using a straight channel constructed by two side plates, as shown in Figure 10a. The channel width was varied from 190mm to 235mm with a step size of 5mm. Here, 190mm is close to the initial radial width of the robot, while 235mm is slightly larger than the maximum radial width after full actuation. During the experiments, the control interval was fixed at ΔT=1s to eliminate the influence of timing variation on the crawling speed.

The representative experimental scenes at path widths of 190mm and 235mm are shown in Figure 10a, and the corresponding speed results are shown in Figure 10b. As the path width increased, the average crawling speed of BAM-STR showed an overall increasing trend. When the channel was narrow, the radial expansion of the robot was constrained by the side plates, which reduced the overall deformation amplitude of the structure. This limited radial expansion also affected the axial contraction, resulting in a smaller effective displacement per gait cycle and a lower crawling speed. As the channel width increased, the robot was able to generate more sufficient radial expansion and axial contraction, thereby increasing the per-cycle displacement and improving the crawling speed.

These results indicate that BAM-STR has path-width adaptability in confined environments. Owing to the compliance of the tensegrity structure, the robot can maintain contact with the channel walls and adjust its axial–radial coupled deformation under different width conditions. This demonstrates that the proposed tensegrity robot not only provides active deformation capability, but also exhibits passive environmental adaptability in confined spaces.

#### 4.3.2. Load-Carrying Crawling Capability

**Figure 11:**
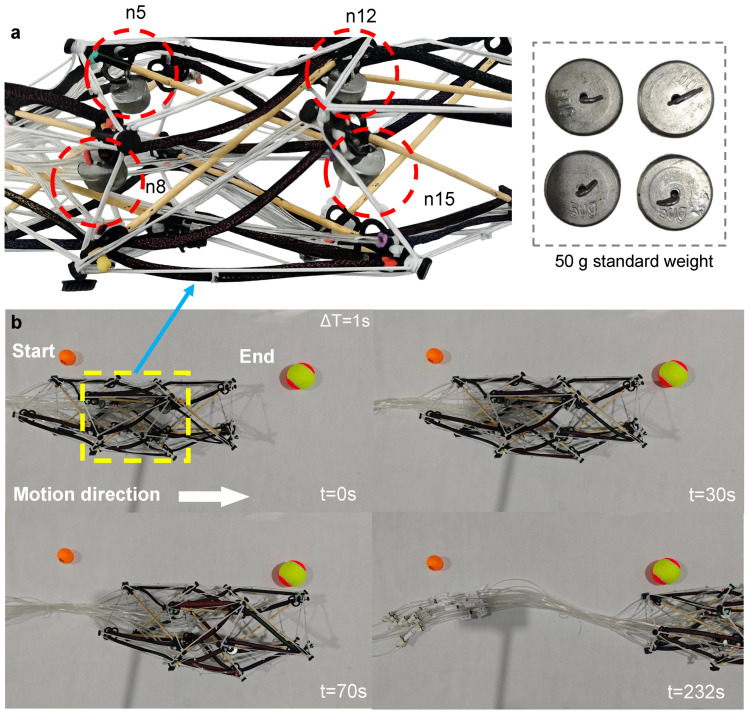
Load-carrying crawling experiment of BAM-STR. (**a**) Arrangement of four 50g standard weights at nodes $n_{5}$, $n_{8}$, $n_{12}$, and $n_{15}$ to achieve a relatively symmetric load distribution. (**b**) Crawling sequence under a total additional load of 200g, with the control interval fixed at ΔT=1s and a 600mm distance between the start and end markers.

In addition to confined-space adaptability, the load-carrying capability of BAM-STR was also evaluated. An additional-load crawling experiment was conducted on a flat surface. In the experiment, four 50g standard weights were attached to nodes $n_{5}$, $n_{8}$, $n_{12}$, and $n_{15}$, as shown in Figure 11a. These nodes are located near the connection region between two adjacent tensegrity units, allowing the additional load to be distributed relatively symmetrically over the robot body. This arrangement can reduce rolling, tilting, and lateral deviation caused by eccentric loading, thereby enabling a more focused evaluation of the forward peristaltic crawling capability of the robot under load-carrying conditions.

During the experiment, the robot crawled on a flat surface, and the control time interval was fixed at ΔT=1s. Two colored marker balls were placed on the test ground to indicate the start and end positions, respectively. The distance between the start and end positions was 600mm. The experimental sequence is shown in Figure 11b. BAM-STR was able to continuously move from the start position toward the end position while carrying four 50g weights. The robot reached the end position at t=232s, completing a crawling distance of 600mm, which corresponds to an average crawling speed of 2.59mm/s. This result indicates that the robot can still generate effective axial–radial coupled deformation under load-carrying conditions, showing a certain load-carrying crawling capability and motion stability.

#### 4.3.3. Ground Adaptability on an Artificial Grass Surface

**Figure 12:**
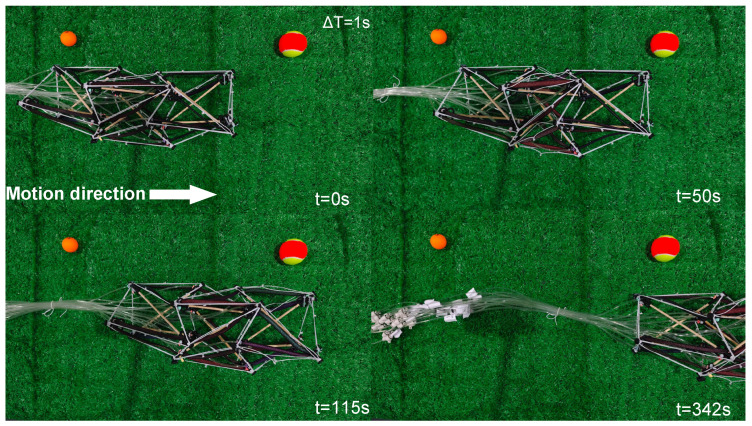
Crawling experiment of BAM-STR on an artificial grass surface.

To further evaluate the ground adaptability of BAM-STR, a crawling experiment on an artificial grass surface was conducted, as shown in Figure 12. Compared with the previously used flat surface, the artificial grass surface has higher roughness and fiber-like protrusions, which increase the contact resistance between the robot and the ground and may cause local interference with the motion of the strut-end components. The experimental setup was the same as that used in the previous experiment.

The experimental sequence shows that BAM-STR was able to continuously crawl forward on the artificial grass surface and finally reached the end position at t=342s. The robot completed a crawling distance of 600mm, corresponding to an average crawling speed of 1.75mm/s. Compared with the flat surface, the frictional resistance and local obstruction caused by the artificial grass surface reduced the forward locomotion efficiency, resulting in a lower crawling speed. Nevertheless, BAM-STR was still able to maintain continuous peristaltic crawling, demonstrating a certain degree of ground adaptability and environmental robustness.

## 5. Discussion and Conclusions

The execution of the BAM-STR task is demonstrated in Supplementary Video S1. BAM-STR proposed in this study combines soft pneumatic actuation, a tensegrity structure, and an earthworm-inspired peristaltic locomotion mechanism to achieve continuous forward crawling under low-pressure conditions. Compared with conventional rigidly actuated tensegrity robots, BAM-STR uses PAMs as active tendons arranged at the diagonal and additional tendon positions, reducing the occupation of the internal deformation space by rigid actuation components. The experimental results show that BAM-STR can achieve approximately 31% axial contraction and 21% radial expansion at an input pressure of 100kPa. This indicates that the proposed structure can convert the axial contraction of the artificial muscles into axial–radial coupled deformation of the overall structure, providing the deformation basis for peristaltic crawling.

From the perspective of locomotion mechanism, the forward crawling of BAM-STR depends on the sequential contraction and release of the three tensegrity units. The bio-inspired segmented peristaltic waveform control strategy enables the robot to generate a deformation wave propagating along the axial direction. This deformation wave works together with the directional friction generated by the anti-slip teeth at the strut ends, thereby producing continuous propulsion. The experimental results show that the control time interval ΔT has a clear influence on the crawling speed. When ΔT=1.0–1.25s, the robot reaches its maximum average crawling speed of approximately 6.5mm/s. This result indicates that an appropriate control time interval can provide a better balance between unit deformation amplitude and gait frequency.

The active tendon actuation mode experiment shows that both the diagonal PAMs and the additional PAMs contribute to structural deformation and forward crawling. When the two types of PAMs are synchronously actuated, the robot obtains better axial contraction, radial expansion, and crawling speed. When only one type of PAM is actuated, the robot can still generate continuous forward displacement. This indicates that the two active tendon groups have a cooperative effect, while the proposed structure also has a certain degree of actuation redundancy and fault tolerance.

The multi-scenario experiments further verify the environmental adaptability of BAM-STR. The robot can maintain continuous crawling within a channel width range of 190–235mm, indicating that its compliant structure can adapt to different lateral constraints. In the load-carrying crawling experiment, BAM-STR completed a 600mm crawling distance while carrying an additional payload of 200g, with an average speed of 2.59mm/s. In the artificial grass surface experiment, the robot also completed a 600mm crawling distance, with an average speed of 1.75mm/s. Although the rough surface increased frictional resistance and local interference, the robot was still able to maintain continuous forward motion, showing a certain degree of motion stability under non-ideal contact conditions.

This study still has some limitations. First, the robot currently relies on an external air source and pneumatic tubes, so its system autonomy and practical deployment capability need to be further improved. Second, hysteresis exists in the PAMs and the overall structure during pressurization and depressurization, which may affect deformation recovery and gait control accuracy. Third, the control time interval ΔT is currently determined through open-loop experimental optimization. However, the optimal time interval may vary under different channel constraints, payloads, and ground contact conditions. Future work can introduce closed-loop sensory feedback, such as pressure feedback, displacement feedback, IMU-based posture information, or soft sensing signals, to evaluate the deformation state and crawling performance of the robot in real time. Based on these feedback signals, ΔT could be adaptively adjusted. More refined pressure control strategies could also be incorporated to improve locomotion efficiency, posture stability, and autonomous adaptability in complex environments.

Overall, the results demonstrate the feasibility of combining McKibben pneumatic artificial muscles, a multi-unit tensegrity structure, and a segmented peristaltic control strategy to achieve continuous crawling under low-pressure actuation. BAM-STR exhibits compliant deformation, stable forward locomotion, and adaptability to different confined and ground-contact conditions.

## Figures and Tables

**Table 1:** Classification and geometric parameters of the three-layer, three-strut tensegrity structure.

Member Type	Number	Length [mm]	Main Function
Strut	9	235	Spatial support
Horizontal string	6	139	Layer shape constraint
Saddle string	12	88	Local stability
Additional string	12	177	Inter-unit connection
Diagonal string	9	182	Torsional stability

## Data Availability

The data supporting the findings of this study are available from the corresponding author upon reasonable request.

## Supplementary Materials

The following supporting information can be downloaded at https://www.mdpi.com/article/10.3390/mi17070857/s1. Video S1: Robotic task execution.
